# GPs’ willingness to prescribe aspirin for cancer preventive therapy in Lynch syndrome: a factorial randomised trial investigating factors influencing decisions

**DOI:** 10.3399/BJGP.2021.0610

**Published:** 2023-03

**Authors:** Kelly E Lloyd, Louise H Hall, Lucy Ziegler, Robbie Foy, Gillian M Borthwick, Mairead MacKenzie, David G Taylor, Samuel G Smith

**Affiliations:** Leeds Institute of Health Sciences, University of Leeds, Leeds;; Leeds Institute of Health Sciences, University of Leeds, Leeds;; Leeds Institute of Health Sciences, University of Leeds, Leeds;; Leeds Institute of Health Sciences, University of Leeds, Leeds;; Translational and Clinical Research Institute, Newcastle University, Newcastle.; Independent Cancer Patients’ Voice;; School of Pharmacy, UCL, London.; Leeds Institute of Health Sciences, University of Leeds, Leeds;

**Keywords:** aspirin, chemoprevention, decision making, NSAID, preventive therapy, primary health care

## Abstract

**Background:**

The National Institute for Health and Care Excellence (NICE) 2020 guidelines recommends aspirin for colorectal cancer prevention for people with Lynch syndrome. Strategies to change practice should be informed by understanding the factors influencing prescribing.

**Aim:**

To investigate the optimal type and level of information to communicate with GPs to increase willingness to prescribe aspirin.

**Design and setting:**

GPs in England and Wales (*n* = 672) were recruited to participate in an online survey with a 2^3^ factorial design. GPs were randomised to one of eight vignettes describing a hypothetical patient with Lynch syndrome recommended to take aspirin by a clinical geneticist.

**Method:**

Across the vignettes, the presence or absence of three types of information was manipulated: 1) existence of NICE guidance; 2) results from the CAPP2 trial; 3) information comparing risks/benefits of aspirin. The main effects and all interactions on the primary (willingness to prescribe) and secondary outcomes (comfort discussing aspirin) were estimated.

**Results:**

There were no statistically significant main effects or interactions of the three information components on willingness to prescribe aspirin or comfort discussing harms and benefits. In total, 80.4% (540/672) of GPs were willing to prescribe, with 19.7% (132/672) unwilling. GPs with prior awareness of aspirin for preventive therapy were more comfortable discussing the medication than those unaware (*P* = 0.031).

**Conclusion:**

It is unlikely that providing information on clinical guidance, trial results, and information comparing benefits and harms will increase aspirin prescribing for Lynch syndrome in primary care. Alternative multilevel strategies to support informed prescribing may be warranted.

## INTRODUCTION

Lynch syndrome (LS) is an inherited condition that increases the risk of developing several cancers, including colorectal cancer.^1^ Aspirin has been investigated as a preventive therapy for colorectal cancer.^2^ The CAPP2 trial observed a reduced risk of colorectal cancer among people with LS randomised to 600 mg aspirin versus placebo at 10 years (hazard ratio 0.65, 95% confidence interval [CI] = 0.43 to 0.97).^3^ In 2020, the National Institute for Health and Care Excellence (NICE) NG151 guideline for colorectal cancer management recommended considering daily aspirin to reduce colorectal cancer risk in people with LS.^4^ NICE did not recommend a dose, but 150–300 mg are commonly used in practice.^4^

Aspirin prescribing is likely to occur in primary care, but GPs may be reluctant to do so.^5^ Ideally, strategies to change clinical practice should be informed by an understanding of the barriers to prescribing behaviour.^6^ An Australian interview study identified several barriers among healthcare professionals about prescribing aspirin for colorectal cancer prevention, including concerns about side effects, limited awareness of the national guidance, and uncertainties about the strength of evidence.^7^ In addition, a large UK survey found GPs who were more aware of aspirin’s cancer preventive benefits were more willing to prescribe the medication to a patient with LS.^5^ In the present study, the relative effects of these different, potentially modifiable, influences on decisions to prescribe aspirin for patients with LS were evaluated in light of the new NICE guidance.

The optimal type and level of information to communicate with GPs was investigated to increase their willingness to prescribe aspirin to a patient with LS. GPs were presented with one of eight versions of a patient vignette, manipulating the presence or absence of three types of information on the effectiveness of aspirin for colorectal cancer prevention:

- existence of NICE guidance (NG151);^4^
- results from the CAPP2 trial;^3^ and
- information comparing the risks and benefits of aspirin.^8^

The main effects of each manipulation on willingness to prescribe aspirin and comfort with discussing aspirin were hypothesised. As exploratory research, two-way and three-way interactions between these main factors on the outcomes were investigated, and barriers and facilitators to prescribing aspirin among GPs examined.

**Table:** How this fits in

National Institute for Health and Care Excellence (NICE) 2020 guidance for England and Wales recommends daily aspirin for colorectal cancer prevention in people with Lynch syndrome, and it is likely that prescribing will occur in primary care. GPs may be reluctant to prescribe because of concerns about the side effects, uncertainties about the strength of evidence, and lack of awareness of the NICE guidance. In a randomised factorial trial, providing GPs with information on these factors did not increase willingness to prescribe, or comfort discussing harms and benefits. Alternative strategies targeting multiple levels of prescribing behaviour among unwilling GPs may support prescribing.

## METHOD

### Setting and participants

GPs in England and Wales were recruited to a cross-sectional online survey. A market research company (M3 Global Research) advertised the survey to their network of over 240 000 GPs. GPs not currently practising and those outside England and Wales were excluded. GPs from Scotland and Northern Ireland were excluded. The stage one registered report was preregistered on Open Science Framework (https://doi.org/10.17605/OSF.IO/B5SFH). CONSORT reporting guidelines were followed.^9^ Informed consent was obtained from all individual participants included in this study, and no confidential information was collected that could identify the participants.

### Experimental design

A 2^3^ factorial trial design was used, with participants randomised evenly across the eight conditions (that is, minimisation) by the survey platform Qualtrics. All vignettes described a hypothetical scenario where a clinical geneticist recommends that the GP prescribes aspirin to a patient with LS (Supplementary Information S1). Three factors were manipulated to form the eight conditions (Box 1). These factors were selected and designed using the authors’ interview data with UK healthcare providers and people with LS (preregistered: https://osf.io/3efg7), the Theoretical Domains Framework,^10^ existing evidence,^5^^,^^7^^,^^11^ and expert opinion from healthcare professionals and a patient representative. The three factors were:

- NICE guidance (NG151) recommending aspirin for people with LS^4^ (versus no information);
- results from the CAPP2 trial investigating the effectiveness of aspirin for people with LS^3^ (versus no information); and
- information comparing the risks and benefits of aspirin^8^ (versus no information).

**Box 1.** Description of the eight experimental conditions (vignettes) in the study and the three factors across the conditions

**Experimental condition**	**NICE guidance (NG151)**	**CAPP2 trial results**	**Risks/benefit information**
**1**	Yes	Yes	Yes
**2**	Yes	Yes	No
**3**	Yes	No	Yes
**4**	Yes	No	No
**5**	No	Yes	Yes
**6**	No	Yes	No
**7**	No	No	Yes
**8**	No	No	No

*NICE = National Institute for Health and Care Excellence.*

Participant blinding was not possible, but participants were only informed about the three factors across the vignettes after survey completion.

### Measures

#### Participant characteristics.

Participants self-reported their gender, status in practice, number of years qualified, and their specialism (see Supplementary Information S2 for the full questionnaire).

#### Willingness to prescribe

GPs were asked how willing they would be to prescribe aspirin to this patient with LS.^11^ Response options ranged from ‘not at all willing’ to ‘definitely willing’.

#### Level of comfort discussing aspirin.

GPs were asked how comfortable they would feel discussing the benefits and harms of aspirin with this patient.^11^ Response options ranged from ‘very uncomfortable’ to ‘very comfortable’.

#### Barriers and facilitators to prescribing.

Participants were asked how much they agree or disagree that 14 factors affected their willingness to prescribe. The factors were based on a similar survey,^11^ with additional items included that were relevant to LS and aspirin. Example factors included the dose of aspirin being prescribed^5^ and the patient’s age.^7^

#### Previous experience.

Participants were asked questions about their professional experience, such as if they have ever prescribed aspirin for colorectal cancer prevention to a patient with LS.

#### Awareness.

Participants were asked if they were aware, before taking the survey, that aspirin can be used to reduce the risk of colorectal cancer, how they first became aware of this, and if they were aware of the NICE guidance (NG151).^4^

### Sample size calculation

The smallest expected main effect size was calculated.^12^ A UK survey of GPs found willingness to prescribe aspirin to patients with LS was as low as 62%.^5^ After considering effect-size data from reviews of interventions targeting prescribing behaviour,^13^^,^^14^ the authors of the current study determined the smallest expected effect size to be a 10% absolute increase in willingness to prescribe aspirin. An increase of willingness from 62% to 72% was calculated as an odds ratio (OR) of 1.58 (∼ Cohen’s *d* of 0.25). With this effect size, power of 90%, α = 0.05, and an equal number of participants per condition, the required sample size was 672 participants. The sample size calculation is available as an R Script (https://osf.io/mgxc4/).

### Analysis

The data are described using proportions and frequencies. The primary outcome was willingness to prescribe, and the secondary outcome was comfort discussing the harms and benefits of aspirin. An ANOVA was used to estimate the main effects and all interactions on the primary and secondary outcomes. Effect coding (−1, 1) was used to enable interpretation of the main and interaction effects simultaneously.^15^

The outcomes of willingness and comfort were also dichotomised at midpoint. Multivariable logistic regression models were conducted assessing the relationship between GPs’ characteristics, awareness, and previous experience on willingness to prescribe (willing versus unwilling), and comfort discussing aspirin (comfortable versus uncomfortable). The proportion of GPs who agreed that each of the 14 factors influenced their willingness to prescribe is also reported.

To minimise missing data, participants were required to answer all survey questions, unless a question was not applicable because of a previous answer. RStudio (version 4.1.2) was used for the analysis, with *P*<0.05 statistically significant. The dataset and analysis scripts were made available on the Research Data Leeds Repository (https://doi.org/10.5518/1184).

## RESULTS

Out of 2200 GPs approached, 867 (39.4%) started the survey. After excluding 195 ineligible participants, 672 GPs were included (Supplementary Figure S1). Recruitment was open between March to April 2022. Table 1 summarises participant characteristics, which were comparable across the eight conditions (Supplementary Table S1).

**Table 1.** Demographic and professional characteristics of the GP sample (*n*= 672)

**Characteristic**	***n* (%)**
**Country**	
England	651 (96.9)
Wales	21 (3.1)

**Gender**	
Female	373 (55.5)
Male	290 (43.2)
Non-binary	1 (0.15)
Another identity	1 (0.15)
Prefer not to say	7 (1.0)

**GP status**	
Salaried/locum GP	389 (57.9)
GP partner	233 (34.7)
GP specialist trainee	44 (6.5)
GP retainers	3 (0.4)
Other	3 (0.4)

**Years qualified**	
0–4 years	24 (3.6)
5–9 years	151 (22.5)
10–14 years	174 (25.9)
15–19 years	143 (21.3)
≥20 years	180 (26.8)

**Specialism**	
Cancer	37 (5.5)
Family history	28 (4.2)
Genetics	4 (0.6)
Preventive medicine	87 (13.0)
Other	132 (19.6)
N/A – no specialty	384 (57.1)

*N/A = not applicable.*

### Awareness of aspirin for colorectal cancer prevention

**Figure 1.**
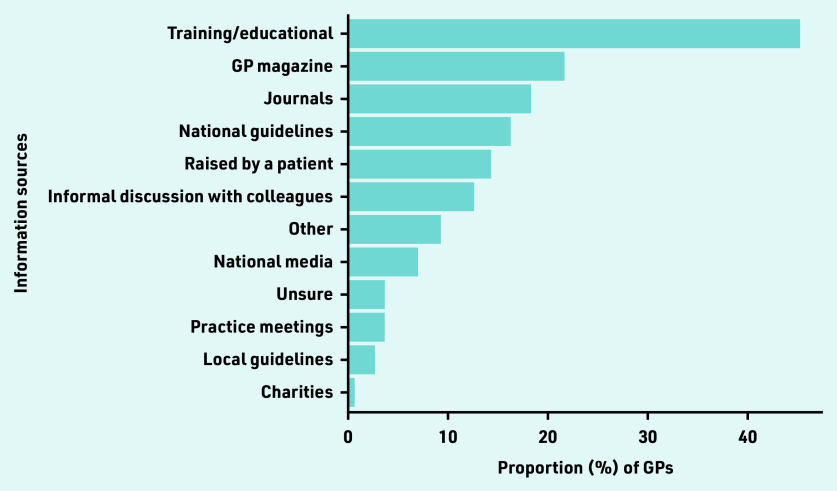
***Proportion of GPs (%) who learnt about the use of aspirin for colorectal cancer prevention in people with Lynch syndrome from various information sources (*n** ***= 300).***

Nearly half (300/672, 44.6%) of GPs reported prior awareness of aspirin for colorectal cancer prevention in people with LS and 17.4% (117/672) were aware of NICE guidance (NG151) recommending aspirin. GPs who were aware of aspirin for LS selected all applicable information sources that made them first aware of using the medication for preventive therapy. The most common sources of information were training days/educational meetings (136/300, 45.3%), GP magazines (65/300, 21.7%), academic journals (55/300, 18.3%), and national guidelines (49/300, 16.3%) (Figure 1). Prior awareness of the NICE guidance was comparable across the eight conditions (Supplementary Table S2).

### Previous professional experience

In total, 46.3% (311/672) of GPs reported previously consulting with a patient with LS and 16.7% (112/672) were unsure. A smaller proportion of GPs recalled having discussed aspirin for prevention (61/672, 9.1% had discussed; 28/672, 4.2% were unsure), or prescribing aspirin to a patient with LS (73/672, 10.9% had prescribed; 40/672, 6.0% were unsure).

### Willingness to prescribe aspirin

Most (390/672, 58.0%) GPs were ‘probably willing’ to prescribe aspirin for the hypothetical patient with LS and 22.3% (150/672) were ‘definitely willing’ to prescribe. In total, 19.7% of GPs were unwilling to prescribe (112/672, 16.7% probably not willing; 20/672, 3.0% not at all willing). Willingness to prescribe among GPs was comparable across the three information components (NICE guidance; CAPP2 results; risk and benefit information) (Table 2). There were no significant main effects or interactions of these three components on willingness to prescribe aspirin (Supplementary Table S2).

**Table 2.** Willingness to prescribe aspirin among GPs presented with each of the three information components (*n*= 672)

**Willingness**	**Total *N***	**NICE guidance, *n* (%)**	**CAPP2 results, *n* (%)**	**Risks/benefits, *n* (%)**
**Definitely willing**	150	80 (53.3)	72 (48.0)	74 (49.3)
**Probably willing**	390	188 (48.2)	194 (49.7)	196 (50.3)
**Probably not willing**	112	52 (46.4)	59 (52.7)	59 (52.7)
**Not at all willing**	20	15 (75.0)	11 (55.0)	8 (40.0)

*NICE = National Institute for Health and Care Excellence.*

In the multivariable logistic regression model, GPs who were unsure whether they had previously prescribed aspirin for colorectal cancer prevention were significantly more willing to prescribe aspirin than those who had not prescribed it; however, confidence intervals were wide (OR 5.67, *P* = 0.032, 95% CI = 1.37 to 34.71) (Table 3). Furthermore, there was no significant relationship between GPs who recalled previously prescribing aspirin and willingness to prescribe (*P* = 0.183). No other factors were associated with willingness to prescribe (Table 3).

**Table 3.** GPs’ willingness to prescribe aspirin by participant characteristics, previous experience, and awareness (*n*= 672)

**Characteristic**	**Willing to prescribe *n* (%)**	**OR (95% CI)**	***P-*value**
**Country**			
England (*n*= 651)	524 (80.5)	1.17 (0.37 to 3.18)	0.771
Wales (*n*= 21)	16 (76.2)	Reference	Reference

**Gender**			
Female (*n*= 373)	297 (79.6)	Reference	Reference
Male (*n*= 290)	238 (82.1)	0.94 (0.61 to 1.46)	0.793
Another identity^a^ (*n*= 1)	0 (0.0)	–	0.994
Non-binary^a^ (*n*= 1)	1 (100.0)	–	0.996
Prefer not to say (*n*= 7)	4 (57.1)	0.27 (0.05 to 1.48)	0.105

**GP status**			
Salaried/locum GP (*n*= 389)	307 (78.9)	1.00 (0.62 to 1.58)	0.988
GP partner (*n*= 233)	193 (82.8)	Reference	Reference
GP retainers^a^ (*n*= 3)	3 (100.0)	–	0.991
GP specialist trainee (*n*= 44)	34 (77.3)	1.22 (0.51 to 3.10)	0.667
Other^a^ (*n*= 3)	3 (100.0)	–	0.992

**Years qualified**			
0–4 years (*n*= 24)	20 (83.3)	Reference	Reference
5–9 years (*n*= 151)	114 (75.5)	0.47 (0.13 to 1.39)	0.205
10–14 years (*n*= 174)	133 (76.4)	0.52 (0.14 to 1.51)	0.263
15–19 years (*n*= 143)	114 (79.7)	0.61 (0.16 to 1.83)	0.409
≥20 years (*n*= 180)	159 (88.3)	1.04 (0.27 to 3.23)	0.952

**Specialism**			
Cancer (*n*= 37)	34 (91.9)	1.75 (0.56 to 7.67)	0.387
Family history (*n*= 28)	21 (75.0)	0.58 (0.23 to 1.59)	0.256
Genetics^a^ (*n*= 4)	4 (100.0)	–	0.989
Preventive medicine (*n*= 87)	69 (79.3)	0.72 (0.39 to 1.38)	0.312
Other (*n*= 132)	104 (78.8)	0.74 (0.44 to 1.26)	0.258
N/A — no specialty (*n*= 384)	308 (80.2)	Reference	Reference

**Previous experience**			
Consulted a patient with LS			
Consulted – yes (*n*= 311)	261 (83.9)	1.57 (0.99 to 2.50)	0.055
Consulted – unsure (*n*= 112)	90 (80.4)	1.23 (0.69 to 2.27)	0.497
Consulted – no (*n*= 249)	189 (75.9)	Reference	Reference
Discussed aspirin with a patient with LS			
Discussed aspirin – yes (*n*= 61)	57 (93.4)	0.81 (0.21 to 3.57)	0.763
Discussed aspirin – unsure (*n*= 28)	23 (82.1)	0.37 (0.10 to 1.53)	0.153
Discussed aspirin – no (*n*= 583)	460 (78.9)	Reference	Reference
Prescribed aspirin to a patient with LS			
Prescribed aspirin – yes (*n*= 73)	68 (93.2)	2.34 (0.72 to 9.05)	0.183
Prescribed aspirin – unsure (*n*= 40)	37 (92.5)	5.67 (1.37 to 34.71)	**0.032**
Prescribed aspirin – no (*n*= 559)	435 (77.8)	Reference	Reference

**Awareness**			
Prior awareness of aspirin in LS population			
Yes (*n*= 300)	261 (87.0)	1.49 (0.91 to 2.49)	0.118
No (*n*= 372)	279 (75.0)	Reference	Reference
Prior awareness of NICE guidance NG151			
Yes (*n*= 117)	107 (91.5)	1.74 (0.80 to 4.07)	0.177
No (*n*= 555)	433 (78.0)	Reference	Reference

*Result in bold is significant.*

a *OR (95% CI) not reported because of insufficient cases. CI = confidence interval.*

*LS = Lynch syndrome. N/A = not applicable. NICE = National Institute for Health and Care Excellence. OR = odds ratio.*

### Discussing the harms and benefits of aspirin

Most GPs felt comfortable discussing aspirin harms and benefits with the hypothetical patient (361/672, 53.7% quite comfortable; 150/672, 22.3% very comfortable), whereas 24.0% were uncomfortable with these discussions (130/672, 19.3% quite uncomfortable; 31/672, 4.6% very uncomfortable). GPs’ comfort discussing aspirin harms and benefits was comparable across the three components (NICE guidance; CAPP2 results; risk and benefit information; Supplementary Table S3). There was no statistically significant main effects or interactions of the components on comfort discussing aspirin (Supplementary Table S3).

In the multivariable logistic regression model, GPs who reported awareness of aspirin for colorectal cancer prevention in people with LS were more comfortable discussing benefits and harms than those who were unaware before the survey (OR = 1.68, 95% CI = 1.06 to 2.72, *P* = 0.031). GPs who were unsure whether they had previously prescribed aspirin were more comfortable discussing harms and benefits than those who had not prescribed aspirin (OR 6.30, *P* = 0.019, 95% CI = 1.61 to 36.67). However, confidence intervals were wide, and GPs who recalled previously prescribing aspirin were not more comfortable discussing the medication (*P* = 0.823). No other factors were significantly associated with comfort discussing aspirin (Supplementary Table S4).

### Factors influencing willingness to prescribe

Among GPs willing to prescribe aspirin, the factors participants agreed were important in their decision were the benefits of aspirin (527/540, 97.6%), the geneticist recommendation to prescribe (492/540, 91.1%), patient interest in using aspirin (491/540, 90.9%), and patient awareness of aspirin harms and benefits (519/540, 96.1%; Table 4).

**Table 4.** The proportion of GPs (%) who agreed that each of the 14 factors influenced their willingness to prescribe (*n* = 672)

**Factor**	**Willing, *n* (%) (*N*= 540)**	**Unwilling,*n* (%) (*N*= 132)**
**Benefits of aspirin**	527 (97.6)	113 (85.6)
**Harms of aspirin**	472 (87.4)	121 (91.7)
**Dose of aspirin asked to prescribe**	455 (84.3)	112 (84.8)
**Prescribing aspirin off-label**	369 (68.3)	110 (83.3)
**Geneticist recommendation to prescribe**	492 (91.1)	93 (70.5)
**Patients’ interest in using aspirin**	491 (90.9)	86 (65.2)
**Patients’ awareness of the harms and benefits of aspirin**	519 (96.1)	104 (78.8)
**Wanting to speak to specialist in genetics before prescribing**	235 (43.5)	86 (65.2)
**Wanting to speak to specialist in colorectal cancer before prescribing**	224 (41.5)	96 (72.7)
**Wanting to speak with another GP before prescribing**	227 (42.0)	74 (56.1)
**Patients’ age**	375 (69.4)	78 (59.1)
**Confidence in aspirin in general**	478 (88.5)	92 (69.7)
**Confidence in aspirin as a form of preventive therapy**	451 (83.5)	104 (78.8)
**Prescribing budget in your practice**	132 (24.4)	28 (21.2)

Those GPs unwilling to prescribe felt the most important factors influencing their decision were the harms of aspirin (121/132, 91.7%), benefits (113/132, 85.6%), dose being asked to prescribe (112/132, 84.8%), and prescribing off-label (110/132, 83.3%).

A higher proportion of those unwilling to prescribe aspirin wanted to speak to a colorectal cancer specialist (96/132, 72.7%) before prescribing than those who were willing (224/540, 41.5%). The patient’s interest in aspirin factored less into the decision making of those unwilling (86/132, 65.2%) than those willing (491/540, 90.9%) to prescribe.

In an open text box, participants were able to write additional factors that influenced their decision. Among unwilling GPs, 12.1% (16/132) suggested that the clinical geneticist should make the first prescription and 7.6% (10/132), that patients should buy aspirin from the pharmacy instead (Supplementary Table S5).

## DISCUSSION

### Summary

In this online factorial experiment, it was found that highlighting the clinical guidance, summarising trial evidence, or giving information on aspirin’s benefits and harms did not increase GPs’ willingness to prescribe aspirin for colorectal cancer prevention.

Reassuringly, most GPs participating in the experiment were willing to prescribe aspirin for a hypothetical patient with LS. However, a fifth of GPs were unwilling. Most GPs who were unwilling described several barriers that behavioural interventions are unlikely to affect, such as the harms of aspirin and prescribing off-label. Alternative strategies targeting multiple levels of prescribing behaviours may be warranted, including targeted support for GPs unwilling to prescribe.

### Strengths and limitations

The study design made it possible to test three different intervention components in a more efficient approach than if individual experiments had been conducted.^16^ However, there are several limitations.

First, the clinical vignette described a hypothetical patient with LS but the specific patient characteristics that may affect GPs’ willingness to prescribe, such as patient age and other medication use, are likely to vary widely among the LS population. The current study only measured GPs’ hypothetical willingness to prescribe aspirin; prescribing behaviour may be different in clinical practice.

The sample of GPs was derived via a market research company and may not be typical of the wider GP community. Finally, a ceiling effect of willingness to prescribe aspirin for preventive therapy may have been encountered, beyond which it becomes difficult to influence the outcome.

### Comparison with existing literature

The study found GPs’ levels of willingness to prescribe aspirin for colorectal cancer prevention to a patient with LS was comparable with a previous cross-sectional UK survey.^5^

Barriers were also observed to prescribing aspirin that were consistent with previous research conducted in breast cancer prevention.

In the current study, several GPs unwilling to prescribe reported a preference for the clinical geneticist initiating the prescription. Similarly, in breast cancer research, GPs have been observed to be more willing to prescribe preventive medicine to a hypothetical patient at higher risk of cancer if a clinical geneticist makes the first prescription.^11^

There are several potential barriers that may prevent aspirin from being initiated in specialist care. Previous UK and Australian research into breast cancer preventive therapy has observed a resistance among hospital-based clinicians to prescribe preventive medicines, given unfamiliarity with prescribing and side effect management,^17^^,^^18^ and lack of access to patients’ medical history.^17^

An Australian study also found that specialist clinicians typically viewed GPs as the main prescribers of aspirin for cancer prevention, while perceiving their own roles as more advisory.^7^

### Implications for research and practice

Multilevel strategies, targeting both patients and healthcare professionals, could be used to support prescribing of aspirin for preventive therapy.

The findings from the current study suggest one approach to supporting GPs’ discussions with patients on the benefits and harms of aspirin for preventive therapy is increasing awareness on using aspirin for this purpose through formal training, educational events, and GP magazines. There may also be scope to change GPs’ knowledge and behaviour through patient-mediated interventions,^19^ as patients were identified as an important information source by many GPs.

One approach to increasing patients’ knowledge is decision aids. This approach has been successful for breast cancer preventive therapy whereby tailored web-based decision aids have been observed to increase patients’ knowledge and to support decision making.^20^^,^^21^ Similar educational tools may also be effective for some patients with LS considering aspirin. In 2020, NICE released a decision aid for people with LS considering aspirin for preventive therapy;^8^ however, its effectiveness on patients’ decision making is unknown.

This study found evidence to suggest that individual guidance and advice from specialist clinicians, especially in colorectal cancer, may help increase the prescribing of aspirin among unwilling GPs.

Local pathways setting out roles and responsibilities of GPs, pharmacists, and specialist clinicians are warranted, and should be clearly described in GP training materials that discuss the use of aspirin for colorectal cancer prevention. Furthermore, these training and educational materials should clarify the role of GPs when asked to prescribe off-label medication, as well as highlighting the importance of ensuring medications obtained over-the-counter are recorded on patients’ medical records.

## Data

The dataset and analysis scripts generated during the study are publicly available in the Research Data Leeds Repository (https://doi.org/10.5518/1184).
